# National Service under New Management

**Published:** 1917-10-20

**Authors:** 


### The Hospital

The Workers' Newspaper of Administrative Medicine and Institutional Life, Administration, National Insurance and Health.

No. 1637, Vol. LXIII. SATURDAY, OCTOBER 20, 1917. PRICE ONE PENNY

### NATIONAL SERVICE UNDER NEW MANAGEMENT.

Sir Auckland Geddes, in accepting the position of Minister of National Service, has undertaken a considerable, a complicated, and a highly patriotic task, and the goodwill of the whole community will go with him in the enterprise. He arrives at a moment when the organisation of National Service finds itself under the shadow of discouragement. An effort has been made, but success has not crowned it. The failure has not been due to any personal fault of his predecessor, but to various circumstances which need not now be recalled. It remains to make a new attempt and to profit by an experience which has not been altogether a happy one. To judge from his speeches the new Minister is bringing to his duties a cheerful and resolute courage and a breezy and bracing sense of humour. He tells us that his *rôle* is a humble one, and that he cannot find anything else to do. Seeing that his obstinate countrymen are set on winning the war he must needs do something, and this is the only opportunity which presents itself. Altogether it looks as if the hour had brought the man, and we may thus be content with the force of circumstances. If the business continues as it has begun it may count on a large success, for all concerned may be glad to co-operate with a chief who displays a temper so engaging and a wit so ready. Co-operation, not compulsion, is to be the motive power, and to this decision the genius of the country will certainly respond. What will be required is to be told in plain terms, and the volunteer will get his opportunity. It will be strange indeed if willing service invited in this spirit does not abundantly justify itself. Not that everything is to be smiles and rose-water. The new Minister has the faculty of the plain word as well as the art of gracious invitation. Somehow or other the national work has got to be done, and those who will not take their share of it are warned that they must not expect a share of the national food. There are hard and narrow times ahead, and each citizen must adjust himself to the situation, and must take his due part of the burden. Superfluous services are to be cut down, for the country needs all available sources of human power and can suffer no invasion of these for the sake of individual convenience and laziness. Three servants are suggested as the maximum even for large households, and no tears will alter the decision. The womenfolk do not escape the traditional compliments, but "young, quite healthy, middle-class femininity" learns that in very large measure it is doing nothing really to help the war along. In all this there is exactly the note which the situation requires, and Sir Auckland Geddes evidently knows well his fellow-countrymen. To oppress them with heavy and laboured exhortations is to invite resistance, but a good-natured scolding with a spice of chaff in it never fails in its appeal. With leading of this order they are ready for any service, and even the laggards will come into line.

The details of the new arrangements relative to the medical profession we do not yet know, but a general sketch of these is announced. The Ministry will have large responsibilities in this direction, for its task, in a word, is the organisation and distribution of the working capacities of the community as these ends are determined by the existence of a state of war. In the forefront, of course, stands the need of men for the fighting forces. But the war is not fought only on the battle front, nor does its triumph depend merely on the provision of men and munitions. A thousand activities must be so continued and coordinated that the life of the nation may be maintained and its will and determination kept steady to the desired end. The whole of this vast field comes under the survey of the Ministry of National Service, and every part of it must be kept in due proportion to the commanding necessities of the situation. Man-power for the Army most certainly, but not in such a fashion or degree as to dislocate other essential demands.

As was foreshadowed some months ago by the Secretary for War, the recruiting problem is to pass out of the direction of the military authorities, and it is the Ministry of National Service which will take up the responsibility. This, of course, means among other duties the provision of civilian medical practitioners for the examination of recruits. The change from military to civil jurisdiction seemed inevitable from the recent Parliamentary inquiry, and we have on previous occasions expressed our sympathy with this policy. But it has yet to secure the justification of success as a practical measure, and skilful organisation and individual helpfulness will be needed to carry it through. The new Minister has called to his aid the existing medical organisations, and he tells us that his advisers include the most competent members of the profession. The country is to be divided into a number of regions, and in each of these is to be appointed a "regional Medical Commissioner," who will be responsible for the working in his district of a new civilian "National Service Medical Board." These Boards will be formed of civilian practitioners prepared to act on a part-time basis, and over each will be a "Deputy Medical Commissioner," who, also, will be a civilian practitioner, but will hold a whole-time appointment. Recruits will no longer be classified in the numerous categories which have hitherto held sway, but will be graded in four groups according to their physical fitness. Careful arrangements are to be made to secure a full opportunity for appeal for any individual who thinks he has not been wisely or fairly treated, and in the last resort such an individual may carry his case outside the National Service scheme and may get the judgment of a medical assessor chosen by the Local Government Board. There is thus complete civilian control, and, in the end, the decision of an official who is completely detached from the recruiting machinery. Such, in brief outline, is the manner in which the recruiting problem is to be tackled, and without committing ourselves to details which are at present unknown, we have no hesitation in saying that the scheme is a promising one. Here, as is the case with laymen, the activities of doctors have to be adjusted to many tasks besides the supply of recruits, and all these activities are to occupy the attention of the medical advisers of the new Minister. Doctors are needed for the Navy, the Army, the Air Service, the Ministry of Pensions, and the Insurance Commission; and last, but by no means least, are the needs of the civilian population. Probably none of these can get anything like an ideal supply, and what the Ministry has to do is to secure such a fair distribution as the circumstances permit. Here indeed is a difficult undertaking, and we shall watch the efforts to discharge it with a critical but by no means an unkindly eye.

